# Investigating the emotional content of older adults engaging in a fall prevention exercise program integrated with dance movement therapy: a preliminary study

**DOI:** 10.3389/fpsyg.2023.1260299

**Published:** 2023-09-18

**Authors:** Michal Pitluk Barash, Michal Elboim-Gabyzon, Einat Shuper Engelhard

**Affiliations:** ^1^The Graduate School of Creative Art Therapies, Faculty of Humanities & Social Sciences, Kibbutzim College of Education, Tel Aviv, Israel; ^2^The Graduate School of Creative Art Therapies, Faculty of Social Welfare & Health Sciences, Emili Sagol Creative Arts Therapies Research Center, University of Haifa, Haifa, Israel; ^3^Physical Therapy Department, Faculty of Social Welfare & Health Sciences, University of Haifa, Haifa, Israel

**Keywords:** fall, fall prevention intervention, older adults, physical therapy, dance movement therapy

## Abstract

Fall prevention interventions for older adults have primarily focused on the physical aspects, overlooking the emotional aspects inherent in falls. This qualitative study presents a novel fall prevention intervention that integrates physical therapy exercise (PTE) based on the Otago Exercise Program with Dance Movement Therapy (DMT) to address the emotional experience during PTE. The aim of this study is to explore the emotional content expressed by older adults during balance-focused exercises and the unique emotional content expressions following the PTE + DMT intervention compared to the PTE intervention. Eleven older adults (aged 81–91 years) from a day center were randomly assigned to either the PTE + DMT group (*n* = 6) or the PTE group (*n* = 5). Interpretative phenomenological analysis of the instructors’ observations and process diary identified three themes that emerged during the sessions: (1) self-image and self-worth, (2) the individual in relation to others, and (3) past memories. These themes highlight both similarities and differences between the groups. These findings provide valuable insights into the emotional experiences encountered by older adults, particularly in the context of falls prevention practices. Recognizing, understanding, and facilitating the expression of these experiences can enhance the effectiveness of fall prevention interventions and contribute to the overall health of older adults.

## 1. Introduction

Falls are a common occurrence in old age, with one out of every three individuals aged 65 and over, living in the community, experiencing at least one fall annually (Montero-Odasso et al., 2022). As life expectancy continues to rise globally, the risk of falls is also increasing. Falls can result in severe consequences, including mortality, morbidity, and physical and psychological injury (Ganz and Latham, 2020). Fall risks factors composed physical factors (such as decreased balance and muscle strength) and emotional factors, such as fear of falling (FOF) and depression.

One of the most significant interventions strategy for preventing falls in older adults is physical exercise, particularly exercises that target balance and functional training (Appeadu and Bordoni, 2021; World Health Organization, 2021). According to research in the field of physical therapy (PT), these exercises have been found to be highly effective, and reducing the risk of falls by approximately 23% (Sherrington et al., 2019; Zhao et al., 2019). The Otago Exercise Program (OEP) is a widely accepted PT program for fall prevention that has been proven effective (Chiu et al., 2021). The OEP consists of muscle strength and balance exercises, walking, and ongoing home practice, and can be practiced independently or in a group setting (Kocic et al., 2018). Group-based OEP has been shown to improve physical indices such as balance, mobility levels, and lower limb muscle strength. Moreover, significant improvement has been observed in these indices and the self-perceived physical health status among participants in group-based OEP compared to those practicing individual home-based OEP (Kyrdalen et al., 2014). However, as the OEP addresses mainly the physical risk factors of falling, such as balance and gait levels, it is suggested that enhancing the program’s effectiveness could be achieved by also addressing the emotional aspect of falling (Lee and Yu, 2020; Pettersson et al., 2020).

Emotional fall risk factors, such as FOF and depression, are prevalent among the older population and can persist in individuals, regardless of whether they have fallen (Hughes et al., 2015; Umegaki et al., 2020; Byun et al., 2021). FOF is associated with various negative outcomes, including decreased physical and cognitive functioning, decreased mental health (Rivasi et al., 2020) and reduced quality of life (Schoene et al., 2019). Research has shown that the FOF among older adults is associated with a tendency to limit daily life physical activities (Fang et al., 2020), and an avoidance of exercise and social activities (Peeters et al., 2020). The association between emotional and physical aspects of the individual can create a detrimental cycle, ultimately increasing their risk of falling. The connection between emotional and physical risk factors (Pauelsen et al., 2018; Umegaki et al., 2020) highlights the significance of integrating emotional aspects into fall prevention programs like the OEP.

Dance movement therapy (DMT) belongs to the mental health professions, and employs experiential interventions and active involvement of the patient to promote mental health and well-being (de Witte et al., 2021). DMT recognize that physical, emotional, cognitive, and perceptual aspects are interrelated in the experience of the self, and that the body and mind are not separate entities (American Dance Therapy Association, 2021; Rosendahl et al., 2021). Thus, DMT centralize the mind–body interconnection as a key therapeutic element, based on established theory and research (Röhricht et al., 2014; American Dance Therapy Association, 2021). The body serves as the foundation for human experiences, as it internalizes, expresses, and shapes emotions, thoughts, and perceptions (Piran et al., 2020; Standal, 2020). Phenomenological models and neuroscientific findings have indicated that bodily sensations and movements, such as postures, gestures, and facial expressions, are inherent components of emotional experiences and implicitly influence daily actions, perception, evaluation of situations, and memory (Fuchs and Koch, 2014; Martin et al., 2016). Studies have demonstrated that body movements and postures can trigger emotions, aid in their identification, and regulate emotional experiences (Shafir, 2016; Koch et al., 2019; Melzer et al., 2019).

DMT uses movement to facilitate emotional, physical, cognitive, and social integration of the individual, without requiring any prior knowledge of movement or physical abilities. In DMT, the therapist helps the patient to processes emotional experiences through body movements and bodily spontaneous expressions, exploring them, sharing the patient’s experience, and discussing it (Shuper-Engelhard et al., 2019; Wengrower and Chaiklin, 2020). The emotional therapist, who is empathetic and adaptable to the patient, facilitates the processing of the emotional experience. This is achieved through the therapeutic relationship established between them, as described in meta-analytic findings that have examined the impact of the therapeutic relationship’s quality on the outcomes of psychotherapy treatments (Norcross and Lambert, 2018).

DMT occurs within the framework of individual or group therapy, using techniques such as mirroring, movement synchronization, and joining the patient’s spontaneous movement and expanding it (de Witte et al., 2021). These techniques have been found to contribute to an increase in empathy (Koch et al., 2019; Christopher and Tamplin, 2022), as well as an increase in feelings of belonging and group cohesion (Chyle et al., 2020). Additionally, the use of shared spontaneous movement can elicit creativity and playfulness, which further contributes to building close relationships (Shuper Engelhard, 2020) and sense of vitality (Koch et al., 2019; Christopher and Tamplin, 2022). Previous studies have shown that DMT can have a positive impact on physiological, functional, and psychological indices among older adults with conditions such as dementia (Ho et al., 2020; Wu et al., 2022), Parkinson’s (Fisher et al., 2020; Wu et al., 2022), and psychiatric disorders (Jiménez et al., 2019). Specifically, DMT has been found to be more effective than physical activity alone in improving daily functioning and reducing depression, loneliness, and negative mood among older people with dementia (Ho et al., 2020).

The aim of this qualitative study is to gain a better understanding of the emotional experiences that older adults may encounter during fall prevention physical therapy exercise (PTE), with the ultimate objective of providing effective therapeutic interventions to address these experiences. Specifically, we aimed to explore (1) the emotional content expressed by older adults following balance-focused exercises of the OEP, and (2) the unique emotional content characteristics demonstrated by a group that had elements of DMT integrated into their OEP exercises (PTE + DMT group) compared to a group who solely practiced the OEP (PTE group).

## 2. Materials and methods

The emotional experience of older individuals during fall prevention interventions remains largely unexplored, unlike the extensive examination of the effects of such interventions on fall risk factors. In order to investigate this emotional experience, a phenomenological-qualitative method was used, considering the novelty of this research area and the limited information in the existing literature concerning the verbal and movement expressions of older adults during these interventions. The choice of the research method stemmed from the desire and aspiration to investigate processes that are largely nonverbal via the subjective experiences of the participants (Cruz and Tantia, 2017). Through an objective description of verbal and nonverbal phenomena, incorporated with the insights of professionals from the fields of PT and DMT, this study intends to provide preliminary and comprehensive information on this under-researched topic.

### 2.1. Study design

This qualitative study is a pilot study and part of a research project aiming to construct and assess a novel intervention model for preventing falls in older adults that integrates PTE and DMT. This study employed a two-group, randomized controlled trial (RCT) design. The current study will be followed by a future RCT that will include a larger sample size consistent of different participants.

### 2.2. Participants

Eighteen participants were recruited for the study at a day center for older citizens through convenience sampling by a social worker who was not involved in the intervention process. Participants were thoroughly screened for eligibility by two physical therapists and the social worker at the day center. The inclusion criteria were: community-dwelling older adults aged 65 years or older; the ability to walk independently for at least 10 m with or without an assistive device; and the ability to walk for 2 min without an assistive device. Exclusion criteria included: heart disease or respiratory disease; a history of stroke, Parkinson’s disease, or any other neurological disorder affecting walking; blindness or deafness that would prevent safe walking and hearing instructions; a vestibular disorder such as vertigo; acute joint or musculoskeletal pain in the lower limbs that would limit continuous walking for 2 min; and significant sensory problems in the lower limbs. Of the initial 18 participants who consented to participate, 11 participants met the eligibility criteria and were considered suitable for the study. None of the participants dropped out during the intervention sessions, except for one participant from the PTE + DMT group who did not participate in the final session.

### 2.3. Interventions- PTE + DMT and PTE

The interventions took place in a 40 m^2^ (430.5 square feet) room suitable for movement, in the day center, with chairs arranged in a circle. Both groups participated in an intervention based on OEP (Campbell et al., 1997; Chiu et al., 2021). Each group took part in six group sessions lasting 40 min, twice a week for 3 weeks.

The intervention included a structured set of exercises increasing in difficulty from week-to-week: a 5 min warm-up, 10 min strength exercises, 20 min balance exercises, and a 5 min cool-down. The PTE + DMT group was given an intervention that integrated OEP-based techniques and DMT techniques to address the emotional experience that arises during physical exercise. This included the encouragement of the participants for spontaneous and creative movement, and an invitation to verbally share the feelings, sensations, and thoughts that arose in the participants following the movement. For example, during an exercise in which participants raised to the tips of their toes from a standing position, the instructor asked them to pay attention to the space between their legs, and to consider what helps them feel stable, as well as what constitutes a safe foundation in life for them. The group was led by a dance movement therapist who was supervised by an expert in the field with over 20 years of experience. The therapist had received training in OEP. The PTE control group received only an OEP-based intervention, which was led by a physical therapist who was supervised by an expert in the field with over 20 years of experience. Both instructors were present in sessions of both groups and were responsible for ensuring the safety of the participants. To ensure the fidelity of the interventions, the instructors went through the sequence of exercises together before each session. Additionally, the instructors received the same number of supervisory hours.

### 2.4. Instruments

#### 2.4.1. Participant characteristics

Self-report questionnaire regarding demographic status and clinical data which included information such as age, marital status, and falls in the past year.

#### 2.4.2. Direct observation

According to Fix et al. (2022), direct observation methods are increasingly utilized in health research studies to gain unique insights into human behavior within healthcare processes. Understanding behavior can be challenging due to its frequently unconscious nature and vulnerability to self-report biases, which rely on participant disclosures. Observation proves particularly valuable in comprehending the experiences of patients, providing an objective perspective from insiders, and facilitating research on topics such as patient-centered care.

In this study, each instructor actively observed the group which they were not instructing and documented descriptive notes based on their open-ended and unstructured observations. These notes encompassed detailed information, including participant presence, verbal statements and feedback expressed by both participants and instructors, and descriptions of movement execution, all of which occurred during the sessions. Each instructor documented each 5-min unit during the session, categorizing the data into two main aspects: participants’ verbal expressions, and their corresponding body movements. Verbally, the instructor documented participants’ responses in relation to the instructor’s instructions and expressions, the executed exercises and movements, their interactions with other participants, as well as any spontaneous statements shared during the session. In terms of body movement, the instructor documented various elements, including the participants’ movements in space, body actions (e.g., walking, sitting, jumping), specific body parts engaged in the movement, body posture (e.g., whether actively upright or supported), movement tempo (e.g., accelerated, quick, decelerated, slow), movement intensity, range of motion (i.e., reach of limbs), flow of movement (free or bounded), frequency of spontaneous and instructed movements, facial expressions, and gaze direction. The instructor also took into consideration the participants’ movement in relation to others, which encompassed the synchronization between the participants, considering both time and rhythm, and the participants’ positional references to other group members (i.e., their distance and body position in relation to one another).

#### 2.4.3. Process diary

The process diary is a research tool used to collect data about the therapeutic process, which was found to be useful for monitoring the progress and development of the therapeutic interventions (Woll, 2013). In DMT, it has been found to facilitate a holistic understanding of the mind–body connection in patients, highlighting the significance of identifying physical expressions to enhance clinical understanding and inform intervention strategies (Bresler Nardi et al., 2023). Serving as a comprehensive documentation tool, the process diary captures the unfolding events during psychotherapy sessions, from the patient’s arrival to the session’s end. Processes of self-reflection and analysis of process diaries, through a combined approach of self-analysis and peer dialog, contribute to the development of critical thinking and the expansion of professional knowledge in therapy (Yu and Chiu, 2019).

In this study, the process diary was documented by the dance movement therapist at the end of each session for both groups. In addition, the diary entries were expanded upon between sessions through mutual conversations between the instructors, incorporating their reflections. The process diary materials included notes about the session’s environment and progression, verbal and movement behaviors, reactions, feedback, and expressions of the participants. In addition, it contained observations about their performance of physical therapy exercises, including any encountered difficulties.

### 2.5. Procedure

All participants were provided with written and verbal information about the study. All participants provided informed consent prior to any evaluation during the enrolment phase. Participants were informed that they had the option to withdraw from the study at any time without penalty or censure. All data collecting and management procedures took the participants’ privacy and confidentiality into account.

Prior to the intervention, participants completed a self-report questionnaire that collected information about their demographic status and clinical data. Afterward, the social worker at the day center employed stratified randomization to allocate eligible participants to one of the two groups, in order to ensure an approximate in mobility ability (i.e., assistive device use) among individuals allocated to each group. In the PTE + DMT group, there were six participants, and in the PTE control group there were five participants. Subsequently, the PTE + DMT group received an intervention that integrated OEP-based techniques and DMT, while the PTE control group received OEP-based techniques only.

### 2.6. Data analysis

Descriptive statistics were calculated for participant characteristics, including median and interquartile ranges. In order to examine the existence of differences in the characteristics between the PTE + DMT group and the PTE group before the intervention, a Fisher’s exact probability test and Mann/Whitney U test analyzes were performed.

The emotional content expressed during group-based physical therapy exercises aimed at preventing falls was analyzed using Interpretative Phenomenological Analysis (IPA) (Smith, 1996, 2004) of the instructors’ direct observations and process diary. This method has been used in previous research by Shuper Engelhard (2020), to examine the stated and implicit significance of structured, free-form dance meetings between grandmothers and their adult granddaughters. The IPA method was selected due to its capacity to offer both descriptive and interpretive analysis, aiming to capture the subjective experiences of the participants (Quinn and Clare, 2008). However, it is acknowledged that the phenomenological analysis generated by the researcher always involves an interpretation of the participant’s experience, which may be influenced to some extent by the researcher’s own beliefs and assumptions. Thus, it is essential for researchers to reflect on pre-existing perspectives that may impact data interpretation (Smith et al., 1999). To minimize this potential bias, the present study involved two additional researchers in the data analysis process.

The data analysis was conducted by the first author, a dance movement therapist and PhD student, and two additional PhD authors, one is an expert in gerontology and DMT, and the other is an expert in gerontology and PT. The IPA involved several stages, with the first two stages performed by the first author. In the first stage, the observations and the diaries were read and re-read several times to become familiar with the content. Afterwards, the data were analyzed by identifying relevant topics, closely aligned with the participants’ own expression, and then divided into clusters. In the second stage, the clusters were reviewed, and a list of themes was compiled. In the third stage, all data, topics, and themes were read and reviewed by the expert authors. In the fourth stage, the first author revised and precisely defined the themes according to the expert authors’ revision. In the fifth stage, the expert authors re-read all the data to ensure that all topics were identified, resulting in a complete list of relevant themes for each topic. In the sixth stage, all authors checked the data, topics, and themes once again to ensure accuracy.

### 2.7. Ethics

The study was approved by the ethics committee of the University of Haifa, Haifa, Israel (Approval 2699).

## 3. Results

### 3.1. Participants’ characteristics

Data from a total of 11 participants aged 81–91 years (median = 85 [82–90] years), were analyzed. Table 1 shows descriptive statistics of participants’ main characteristics in each group, including age, gender, marital status, whether they live alone, falls in the last year, and use of an assistive device. No statistically significant differences were identified between the groups in main baseline characteristics (demographic and clinical variables).

**Table 1:** Descriptive statistics of participants’ main characteristics.

Characteristics	PTE + DMT group (*n* = 6)	PTE group (*n* = 5)	*p*-value
Age (years)	86.5 (81.5–91)	85 (81.5–87)	0.52
Gender			
Female	6	4	0.45
Male	0	1	
Marital status (*n*)			
Marital relationship	0	2	0.18
Divorced	1	0	0.55
Widowed	5	3	0.36
Living alone (*n*)	6	3	0.06
Falls in the past year (*n*)	3	4	0.3
Assistive device (*n*)	2	2	0.45

Values are presented as median (interquartile range) or *n*. PTE, physical therapy exercises; DMT, dance movement therapy.

### 3.2. The emotional content elicited by balance-focused exercises

**Figure 1:**
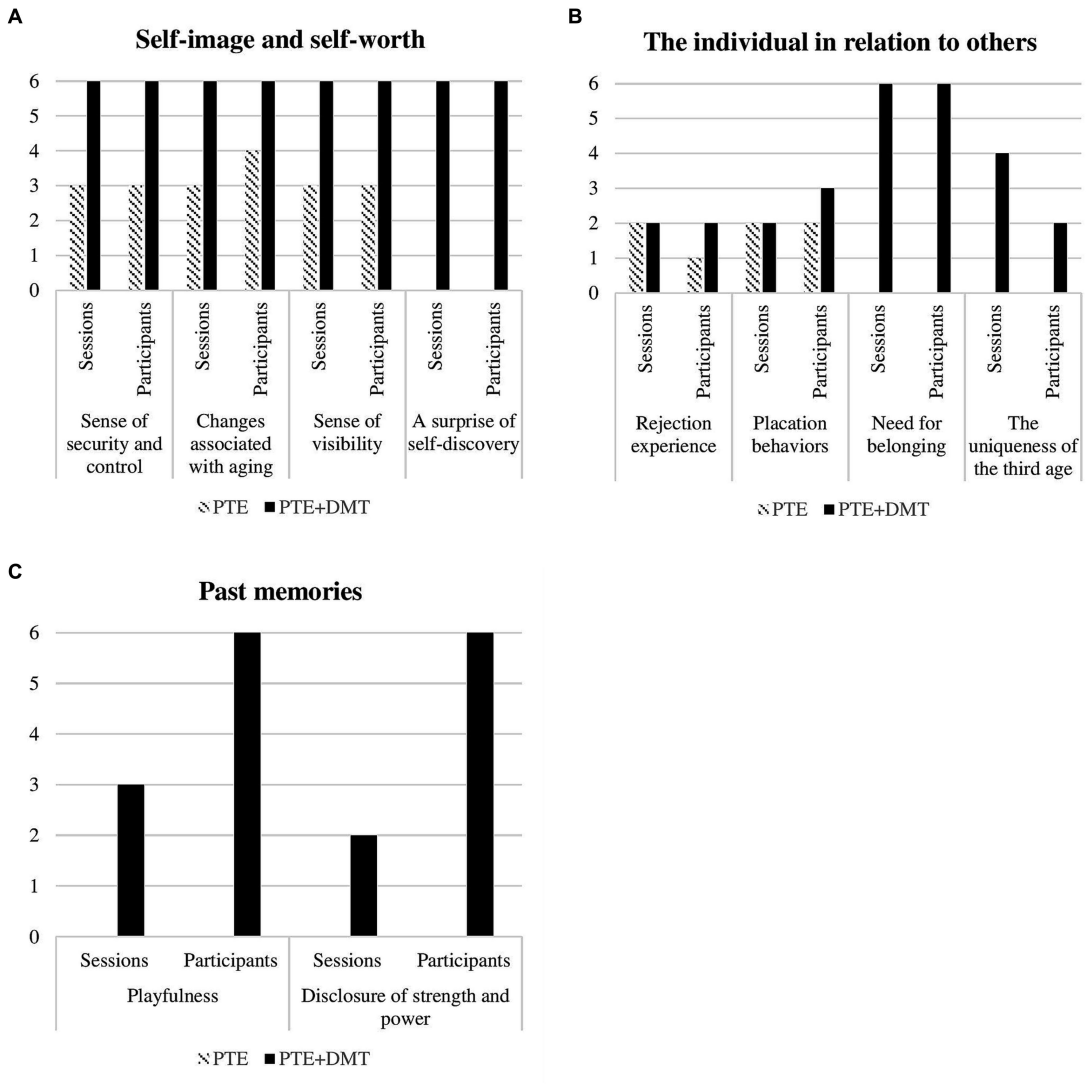
Differences between the PTE + DMT group and the PTE group in emotional contents elicited by fall prevention physical exercise. The themes: **(A)** self-image and self-worth, **(B)** the individual in relation to others, and **(C)** past memories. Sessions = The number of sessions in which the emotional content was presented out of a total of 6 sessions. Participants = The number of participants who expressed the emotional content out of all the participants in each group.

Analysis of the present study revealed three themes that emerged during the sessions: (1) self-image and self-worth, (2) the individual in relation to others, and (3) past memories. Each theme consisted of subthemes that encompassed both verbal and movement elements (see Table 2). The themes highlighted the differences and similarities between the PTE + DMT group (*n* = 6) and the PTE group (*n* = 5), as observed through the occurrence of themes, their frequency, and the number of participants expressing them during the sessions (see Figure 1).

**Table 2:** Emotional content elicited by fall prevention physical exercises.

Themes	Subthemes	Movement expressions
Self-image and self-worth	Sense of security and control	Spontaneous movement
		Upright posture
		Intensity
		Rest
	Changes associated with aging	Comforting touch and movement
	Sense of visibility	Extraverted movement
	A surprise of self-discovery	Stability
		Acceleration
The individual in relation to others	Rejection experience	
	Placation behaviors	Performing instructed movement
	Need for belonging	Eye contact
		Smiling
		Changes in body posture and placement
		Synchronization
	The uniqueness of the third age	
Past memories	Playfulness	Indulging movement
	Disclosure of strength and power	Fighting movement

#### 3.2.1. Theme – self-image and self-worth

Following interventions aimed at enhancing balance, all participants in the PTE + DMT group (*n* = 6) expressed their perceptions regarding their personal qualities and abilities in every session. In the PTE group, half of the participants (*n* = 3) did so in half of the sessions. In both groups, participants reported a heightened sense of security and control over themselves and their surroundings, acknowledged the changes associated with aging, and expressed a need for a sense of visibility in the group. Moreover, participants in the PTE + DMT group only, expressed surprise at self-discovery.

##### 3.2.1.1. Sense of security and control

All participants in the PTE + DMT group expressed their sense of confidence, control over their bodies, and independence in all sessions. Some of their expressions included statements such as, “The desire to do something gives me confidence. When I move and dance, I am balanced, I can dance all day long,” “I do not need support from the chair,” and “Let us swing the balance.” In addition, participants demonstrated a sense of confidence in **spontaneous movements** that challenged their balance (*n* = 4). For example, one participant twirled a walking stick in the air as if she were performing a cabaret dance or military movements, while another swung her leg while standing sideways. Two participants were observed walking with **upright posture** for an extended period of time while remaining light on their feet and keeping their gaze focused on their surroundings. In addition, one participant walked on tiptoe without support.

Confidence in the body was expressed through **intense movements**. At the time of the study, the local atmosphere was characterized by an unsettling sense of political and security instability. During this time, all participants in the PTE + DMT group spontaneously made direct and sharp fist movements emanating from the center of the body while loudly making statements such as, “Beware of me,” “Those fooling around should be careful,” and “We will overcome danger.” In addition, in the final session, one participant energetically and quickly demonstrated how she stood up: “I am a hero, I can stand up and sit down.” The body was also a safe haven for relaxation, as one participant fell asleep while sitting and relaxing her body. At the end of the last session, a participant in the PTE group pointed out the importance of mental thinking in feeling safe: “A lot depends on your head – you think you are about to fall, and you gain the confidence that you are not going to fall.”

Another expression of confidence was evident in both the PTE + DMT group (*n* = 5) and the PTE group (*n* = 2) when leading movements and instructing the group. For example, in the PTE + DMT group, participants took the lead in instructing the group’s movements (spontaneous or instructed movements): “Now get on your heels,” “Pat,” “Now I am going to tell you what to do,” “Make circles,” “Change legs,” and “Right to left.” Similarly, participants in the PTE group demonstrated their control over the group by correcting each other’s movements (instructed movements) by saying, “You are not doing it right (while demonstrating the movement)” and “Here, do it this way (while demonstrating the movement).”

Routine and certainty provided participants in both the PTE + DMT group (*n* = 6) and the PTE group (*n* = 5) with a sense of security. Participants in the PTE + DMT group (*n* = 4) expressed a desire for consistency in seating arrangements: “Same seats as last time,” “Everyone in their seat,” “Do not change,” and “Assigned seats.” Upon entering the room, participants in both the PTE + DMT group (*n* = 4) and the PTE group (*n* = 2) scanned the room to determine who was present: “Who is missing?” “Did she not come today?” and “She is not here today.”

Only in the PTE + DMT group, some participants (*n* = 3) told of morning habits that helped them feel more in control: “I get out of bed, first thing I do is get everything in order, then I get in the shower, take a shower, and pick out my clothes,” “I prepare (the clothes) the night before,” and “We do not come here (to the day center) until the house is in order.” In addition, repeated practice of standing up from a seated position, along with educational instruction on correct and safe technique, resulted in increased confidence in standing up at the fourth and final session (*n* = 4).

Only in the PTE + DMT group, when suggestions were given during the sessions to **rest**, pause, and explore what promotes a sense of security and stability in the body, the participants were able to experience a sense of control. In all sessions, participants (*n* = 4) chose to pause or sit down after engaging in strenuous or challenging movements, and they also utilized a chair for support while standing. They adjusted their movements based on their ability to maintain stability and did not always adhere strictly to the given instructions. For example, one participant moved her leg to the side instead of maintaining a static position, while another shifted from one leg to the other in the middle of a movement when feeling fatigued. During the final session, participants (*n* = 3) expressed that they possessed control and confidence beyond the sessions, stating, “I did not exercise today, I preserved my strength for this,” “It liberates and instills confidence that you can practice it at home,” and “I will continue everything at home… I will do what is suitable and leave out what is not suitable.”

All participants in the PTE + DMT group only, explored how their movements and body positions contributed to a sense of security, stability, and body control. Specifically, they paid attention to the base of standing and the space between the legs (*n* = 2), the tilt of the back when standing on tiptoes (*n* = 5), the support of the hands when rising from a chair (*n* = 5), the position and height of lifting one leg in the air (*n* = 5), and the sensation of the feet touching the ground (*n* = 1). For example, one participant stated, “I feel stable when my base is wider,” while another noted, “You can lean on your knees,” and another commented, “All my toes are on the ground.”

Only in the PTE + DMT group (*n* = 2), questions that guided participants in finding alternative ways to regain control contributed to a positive experience of “existence” when they expressed a lack of control. For example, when one participant expressed that her legs were not functioning, she was asked what was functioning and she responded, “the shoulders” and began to move them. Another participant felt less tall than the instructor when she stood on her toes but noted that in her internal experience it was possible to feel taller.

Both the PTE + DMT group (*n* = 5) and the PTE group (*n* = 2), exhibited expressions of low confidence in their bodies and lack of control over them. The difficulty in moving different body parts and immobility were expressed by statements such as: “I can not stand on this leg,” “I have a problem with my shoulder,” and “My knees are not functioning.” Participants also shared experiences of losing control of the sphincters when waking up or frequent urination. Bodily sensations were experienced as uncontrollable, with participants stating, “Why does it hurt?” and “Why are my legs shaking?”

Only in the PTE + DMT group, the impact of the environment on the feeling of security and control was significant. During the exercises, participants expressed feelings of lack of control related to the fact that their therapists were exerting control over them (*n* = 3), with participants stating, “They tell us to do this and that,” “They tell us what to do,” “They transferred me from an art meeting,” and “I came here because I was forced.” Additionally, participants shared that the location and community influenced the level of security (*n* = 2). One participant said, “I am alone at home, so I do not have the confidence (to exercise),” and another, “It is challenging to leave the house, except for the day center because it is my home.” Emotional sharing following a security incident was also common in the PTE + DMT group (*n* = 5), with participants expressing, “I did not fall asleep,” “I could not continue watching the news… yesterday I was distressed,” and “It is scary in this situation.”

##### 3.2.1.2. Changes associated with aging

Participants expressed verbally and through their movements a sense of loss of physical function associated with aging. In the PTE + DMT group, participants, while sitting supported in a chair, shared their experiences of the body no longer functioning, frequent sleep disturbances, and limitations in physical movement (*n* = 5). During prolonged and strenuous movements, all participants in the PTE + DMT group alternately sat down, explaining that they had experienced a health incident or fall in the past, and expressed fatigue, stating “we got tired.” In the PTE group, participants also shared a change in their physical abilities when performing exercises (*n* = 4), stating things such as “My steps have gotten small with age” and “What you (the instructor) say helps me, but it’s hard for me… time takes its toll.”

In the PTE + DMT group only, all participants expressed self-compassion and self-acceptance of their abilities, particularly after engaging in high-intensity movements. Self-compassion and self-acceptance were demonstrated through **comforting touch and movements** involving deep touch pressure, caressing the body, and even pausing movement during exertion in response to the instruction “I am allowed to stop.” Verbal expressions accompanied the movement expressions. For example, during a knee massage, a participant said, “Let us pamper ourselves,” and during a self-hug, another participant said, “To love ourselves.” When choosing to sit down during a challenging exercise, one participant shared, “I am content with who I am.” During another challenging exercise, a participant stated, “There is one who can do more and another who can do less.”

In addition, participants in the PTE + DMT group (*n* = 3) emphasized changes and losses in their family circle, which were expressed during comforting self-touch such as hugging or caressing: “I used to hug my grandchildren, but now there’s no one to hug,” “If I am not for myself, who will be for me?” and “There is no one to hug her (one participant referred to another), so she hugs herself.” Other changes were related to their lifestyle, such as prolonged sitting during the day (*n* = 2), and a perceived lack of qualities typically associated with youthfulness, such as lightness, playfulness, and vitality (*n* = 2). These reflections were expressed following spontaneous dance movements and group singing during the sessions.

In the PTE + DMT group only (*n* = 5), the changes associated with advancing age were expressed as a split between the body and mind. Throughout most of the sessions, participants expressed a sense of disconnect between their head, body, and mind (*n* = 5). During strenuous movement, participants stated: “The mind is young,” and one participant expressed that she can only imagine being young. After sharing the body’s difficulty in performing movements, participants said: “The head works,” “The mind is fine, but my physical health is less so.” Other perceptions related to body image were expressed in the description of the effort associated with moving the body (*n* = 3): “Working with the hands,” “If we do not move, we will degenerate.”

In the PTE + DMT group only (*n* = 5), participants expressed feelings of jealousy and comparison towards the instructors, along with sharing experiences of loss and changes associated with old age. Jealousy related to the physical aspect was expressed in the first session after a long sequence of standing movements when a participant said, “You (the instructors) are young, it’s hard for us.” As the sessions progressed, some participants expressed curiosity about the instructors’ frequency of physical activity (*n* = 3), while others expressed astonishment at their movements (*n* = 2), stating, “You are sitting upright… look how upright you are” and “How tall you are.” During an invitation for deep touch pressure, a widowed participant said, “Let your husband give you a massage.”

##### 3.2.1.3. Sense of visibility

A desire to be seen by the instructor and group members emerged in various verbal and non-verbal forms. In the PTE + DMT group, participants explicitly stated (*n* = 2): “Look, I can do it without holding!” and “This is how I am doing it now,” accompanied by a demonstration of the movement. In the PTE group, participants sought feedback from the instructor regarding their exercise performance (*n* = 3), and one participant explicitly requested the instructor to observe her during exercise.

Non-verbal expressions of **extraverted movement** were also observed in the PTE + DMT group only (*n* = 5), to draw attention. These included expansive movements, such as lifting both legs straight in the air while seated or extending arms to the sides. Participants created sound through movements like clapping hands on hips or stomping feet on the floor, and they intentionally changed their positioning within the space, such as entering the center of the circle during a dance movement. Furthermore, participants showcased their proficiency to the instructor by performing demanding movements, such as waving hands while standing on tiptoes or balancing on one leg without support (*n* = 3).

##### 3.2.1.4. A surprise of self-discovery

Only in the PTE + DMT group, all participants expressed surprise at their own abilities when engaging in novel and challenging movements and directed attention to physical sensations. Through exploring different body parts and postures, participants discovered movements that were surprisingly easier for them to perform (*n* = 3): “I find it easier on the left,” “It’s actually easier to lean back.” Furthermore, when participants were encouraged to engage in balance-challenging movements and experienced success, it was manifested in their enhanced **stability**, such as a prolonged maintenance of posture, or self-initiated **acceleration** of movements (*n* = 5). For example, during the repeated movement of rising from a seated position, participants were observed accelerating their standing throughout the sessions, despite testifying that it was a difficult movement. While standing, they raised their heads and had a smiling facial expression.

In the PTE + DMT group, participants were surprised by their physical abilities during unstructured spontaneous movement. Circular movements of the pelvis and shoulders reminiscent of belly dancing amazed group members with the quality of their performance, leading to mutual compliments (*n* = 4), despite one participant’s initial expressed skepticism: “We are past the days of belly dancing.” Another participant offered a wavy and repetitive hand movement, and exclaiming, “We are soaring, like birds.”

In addition, during structured movements, one participant discovered her endurance and requested an increase in the movement difficulty level, stating, “My blood is flowing, it wants more,” and “We are not being exerted as much as you say.” Another participant was surprised by her speed when she arrived first at the session, despite other group members having gone ahead of her towards the session room. However, one participant expressed doubt about her abilities, testifying “I can not” before attempting to perform a sequence of movements demonstrated by the instructor.

#### 3.2.2. Theme – the individual in relation to others

In both groups, content related to the experience of rejection had emerged (PTE + DMT group: *n* = 2; PTE group: *n* = 1), and behaviors of placation were observed (PTE + DMT group: *n* = 3; PTE group: *n* = 2), in a minority of sessions. Only in the PTE + DMT group (*n* = 6), all sessions had content related to the need for social belonging, and a minority of sessions had content related to the experience of the third age uniqueness (*n* = 4).

##### 3.2.2.1. Rejection experience

Both groups discussed experiences of rejection in interpersonal interactions, both within and outside of the group (PTE + DMT group: *n* = 2; PTE group: *n* = 1). In the PTE + DMT group, a participant shared that when she was diagnosed with COVID-19, others in the center distanced themselves from her and stigmatized her. Another participant attended the sessions only after receiving a personal invitation from the instructor, emphasizing the significance and value attributed to her presence and inclusion within the group. During a session where some participants hugged and danced together, the same participant stated, “I have already been in the middle enough, now I want to be on the side.” In the PTE group, a participant shared upon entering the session room, that she was told that the group was not suitable for her.

##### 3.2.2.2. Placation behaviors

Participants in both groups complied with the **instructed movements** due to their desire for a sense of belonging (PTE + DMT group: *n* = 3; PTE group: *n* = 2). In the PTE + DMT group, one participant remarked, “We are disciplined” when the group members arrived on time for the session and when recalling the steps of safely getting up from a chair as instructed. The same participant reiterated this statement while slowly rising from a seated position when the group was asked to synchronize their movements, and another participant added, “We are like soldiers.” During an exercise, one participant expressed discomfort due to pain but stated her intention to continue with the exercise. In the PTE group, a participant complained about knee pain and explained that she would be unable to practice, but continued to try, like the other participants.

##### 3.2.2.3. Need for belonging

In the PTE + DMT group only, all participants engaged in various movement expressions, including prolonged **eye contact, smiling**, and **changes in body posture and placement** in relation to other participants and the instructor. Furthermore, participants expressed affection through movements, such as hugging and blowing kisses to others in the group. Participants also created **synchronization** in their movements during the sessions, such as patting their hips to create a common rhythm (*n* = 6), clapping that led to singing together (*n* = 6), repeating standing and sitting movements while breathing (*n* = 5), and moving in the same direction when facing each other (*n* = 4). In contrast, one participant moved in the opposite direction of the person in front of her, looked down, and halted her movement midway. Other instances of this participant distancing herself or avoiding contact were observed, such as choosing to sit during some movements or refraining from movement altogether. This participant did not attend the final session.

Throughout the sessions, participants engaged in twisting movements while maintaining eye contact with each other in a circle formation. As the sessions progressed, most participants (*n* = 5) began to greet each other in a light-hearted manner, using phrases such as “Good morning,” “Good afternoon,” and “Good night.” Participants also complimented each other during their movements (*n* = 4) with comments such as “I enjoy watching you,” “You are doing a great job,” and “She is perfectly fine.” Notably, when one participant expressed the need for a hug, another participant approached and embraced her, saying “I will give you a hug.” At the end of a session, a participant shared a movement that she found enjoyable, and another participant joined in and expressed the same sentiment, stating “For me too.”

##### 3.2.2.4. The uniqueness of the third age

Only in the PTE + DMT group, the majority of participants (*n* = 4) expressed unity in the last two sessions regarding their positive experiences and unique qualities in comparison to younger generations. These sentiments emerged during spontaneous movements and singing that represented childhood games, accompanied by comments such as “young people do not even know these games,” “They are laughing at us,” and “Our children are unaware of this.” Participants also discussed their diligent morning routines, stating to the instructor, “It is not like your generation,” and “From generation to generation less is done.” Furthermore, participants shared that they were well-behaved children who respected their parents and added, “Today’s generation is rude.”

#### 3.2.3. Theme – past memories

Only in the PTE + DMT group, movements that evoked imagery triggered associations with the participants’ past experiences. In half of the sessions, these associations were expressed through playful behavior (*n* = 6), while in a minority of the sessions, participants displayed disclosure of strength and power (*n* = 6).

##### 3.2.3.1. Playfulness

Among all participants, movements requiring balance developed spontaneously and transitioned into **indulging movements** reminiscent of childhood games, children’s songs, and joy. For instance, all participants began shaking their legs while sitting, which extended into swaying their legs back and forth. Recognizing the childlike nature of their movements, participants laughed and began singing a children’s song. Later, a participant shared an energetic memory of enjoyment and ability, recalling running to school as a child.

In another case, standing on one leg transitioned into jumping, which triggered memories of childhood games among all participants, such as hopscotch, jump rope, and hide and seek. Similar games were also recalled after a sequence of standing up and sitting down, leading to the group singing a childhood song together. During another session, where standing up from seated position evolved into singing, most participants (*n* = 5) spontaneously clapped their hands, danced, and skipped while singing a folk song from their childhood, entering and leaving the circle. The movements and words had a drifting and flowing qualities, so the instructor had to initiate the end of the singing and movements. All participants smiled, and some (*n* = 2) shared their experience: “There was laughter” and “It was a joy of life.”

##### 3.2.3.2. Disclosure of strength and power

In the final two sessions, when memories of strength were brought up, the participants spontaneously moved into **fighting movements**, and the opposite – fighting movements evoked memories. For example, during a discussion of childhood games, a participant spontaneously stood up with vigor and used hand gestures to mimic throwing, as in a game of five stones. Another participant enthusiastically shared a story about running to a bus and holding its doors for latecomers. Another participant demonstrated a quick walk around the chair. Similarly, after prolonged standing, performing directional and strong hand movements and patting the body, participants expressed feelings of strength, which evoked memories of pregnancy and childbirth (*n* = 4). One participant shared how she carried ice to the refrigerator while being 9 months pregnant, while another participant shared the long journey she undertook with her husband to give birth. In the final session, expressions of power and ability intensified, with one participant making a fist movement, another demonstrating standing up without support from a chair, and participants declaring, “We are heroes,” and “We are not afraid.”

## 4. Discussion

The current study sought to explore the emotional experiences among older adults participating in fall prevention PTE. The findings indicated that the physical exercises associated with falls elicited emotional responses pertaining to various aspects of aging, including the themes of “self-image and self-worth” and “the individual in relation to others.” Notably, the PTE + DMT group exhibited an additional theme of “past memories” and subthemes of “a surprise of self-discovery,” “need for belonging,” and “the uniqueness of the third age” that encompassed positive emotions and perspectives. Overall, all the themes were more frequently observed in the sessions and involved a greater number of participants in the PTE + DMT group compared to the PTE group. The discussion will be divided into two parts. The first part will explore the emotional experiences that emerged during the OEP in general. The second part will focus on the unique emotional content that specifically rose in the OEP that included integrated elements of DMT.

### 4.1. The emotional content expressed by older adults during fall prevention physical exercise

The results of our study highlight the importance of the self-image and self-worth theme in the context of balance-focused exercises for older adults. This finding may be related to the tendency of self-esteem to decline in old age, especially in individuals over the age of 90 (Orth et al., 2018). Furthermore, physical activity has been shown to improve self-esteem and self-efficacy in older adults (O'Neill and Forman, 2020), which may explain the emergence of this theme in the interventions. The bidirectional relationship between physical activity and body image, encompassing an individual’s subjective perception of their physical appearance and functioning (Vani et al., 2021), may also contribute to the salience of the self-image theme in the sessions. For older adults, the functional aspect of body image holds particular importance due to its impact on the cognitive, emotional, and behavioral management of the aging body (Bennett et al., 2017).

In the context of falls, our intervention shed light on the significance of self-image and, specifically, self-confidence in old age. Falls can have a negative impact on self-image and self-confidence, even when they do not cause physical harm (Hartholt et al., 2011). Furthermore, fear of falling can lead to reduced physical activity and exercise avoidance (Fang et al., 2020; Peeters et al., 2020), leading to a decline in self-image and perpetuating a cycle of reduced physical activity (Sabiston et al., 2019). These findings underscore the need to acknowledge the impact of falls on self-image and self-worth that may arise during fall prevention exercises and address them effectively.

Another prominent theme that emerged in both groups was “the individual in relation to others.” This finding is not surprising, as old age brings several changes in the stable course of an individual’s life that affect their social connections, and increase the risk of social isolation and loneliness (Ayalon, 2018). These changes may include the loss of friends and a spouse, retirement, functional limitations such as reduced mobility, and declines in physiological and cognitive health (Donovan et al., 2017; Merchant et al., 2020). Notably, the majority of participants in the present study were widowed females living alone, placing them at a higher risk of loneliness, depression (López Doblas and Díaz Conde, 2018), and falls (Appeadu and Bordoni, 2021) due to these characteristics. This study emphasizes the complexity of physical activity interventions aimed at fall prevention, as they elicit emotional experiences associated with age-related social changes and their consequences.

The current findings highlight the importance of social interaction during group exercises. The findings are consistent with previous research demonstrating the impact of group physical activity on social aspects such as social support, which has been associated with increased physical activity participation and adherence in older adults (Zimmer et al., 2022). Additionally, studies have demonstrated that a lack of social support is associated with apprehension and fear towards adverse events like falls (Naseri et al., 2020), and that social relationships and involvement are crucial for the mental health and well-being of older adults (Keisari et al., 2020). However, it is essential to note that not all social experiences are positive, and the quality of interactions plays a critical role in determining whether beneficial interactions occur (Feeney and Collins, 2015). Indeed, in the present study, various aspects of social experiences were observed, encompassing both positive elements such as the need for belonging and the uniqueness of the third age, as well as negative elements including rejection experiences and placation behaviors.

### 4.2. Unique emotional content in fall prevention exercise addressing emotional experiences

During the sessions, the PTE + DMT group presented unique emotional content that emerged during balance-challenging exercises. Furthermore, this group exhibited a greater diversity and frequency of emotional expressions compared to the PTE group. These findings can be attributed to the inclusive nature of DMT, which encourages participants to engage in spontaneous and creative movements beyond the structured exercises. In DMT, individuals are invited to share their imagery, feelings, sensations, and thoughts associated with the movements, and also to participate in the collective movement experiences within the group. This stands in contrast to an authoritative structured instruction that primarily focuses on repetitive exercise routines. These therapeutic elements serve to encourage individuals to explore and tap into their inner resources, thereby fostering experiences of renewal and the development of mental resilience in old age (Capello, 2018). Mental resilience has been suggested as a potential means to reduce FOF (Dolan and Pool, 2023).

The subtheme of “a surprise of self-discovery” was uniquely observed in the PTE + DMT group. This finding aligns with a previous study that explored dance interventions based on DMT principles among grandmothers aged 80 to 95 and their granddaughters (Shuper Engelhard, 2020). The findings of that study demonstrated that engaging in dance activities involving movement mirroring, spontaneous expression, and creativity elicited a sense of surprise among the grandmothers as they discovered newfound abilities in their bodies, such as strength and power, despite experiencing movement difficulties. In addition, the current finding is also consistent with the results of our previous study, which indicated a trend towards improvement in the physical components of self-perceived health status in the PTE + DMT group, but not in the PTE group (Pitluk Barash et al., 2023).

The discovery of the body’s capabilities through movement aligns with empirical evidence demonstrating the positive impact of DMT interventions on physical abilities. Studies that investigated the impact of dance-based interventions on fall risk factors among older adults, incorporating movement elements present in DMT such as spontaneous movement, rhythm, and synchronization, have reported improvements in physical risk factors for falling. These include enhanced mobility functions and endurance in healthy older individuals (Liu et al., 2021), as well as improved balance and gait abilities among older individuals with health conditions (Hackney et al., 2013; Ismail et al., 2021; Hewston et al., 2023).

Regarding the theme of “the individual in relation to others,” positive expressions were exclusively observed in the PTE + DMT group, specifically with regards to the subthemes of “need for belonging” and “the uniqueness of the third age.” It is noteworthy that despite group physical activity among older adults being known to reduce loneliness (Li et al., 2023; Paquet et al., 2023) and facilitate social belonging (Morrison et al., 2023), related expressions did not emerge in the PTE group. The current findings can be explained by the therapeutic change factors of DMT, which emphasize inviting participants to engage in synchronized movements, create a common rhythm, and move together (de Witte et al., 2021).

Synchronization of body movements between participants was a key movement expression observed only in the PTE + DMT group. It involves aligning movements in terms of time and rhythm (Rabinowitch, 2023), representing social unity (Lakens, 2010). The current results are consistent with previous studies among older adults, indicating that synchronized movement promotes connectedness, reduces loneliness (Abu Elheja et al., 2021) and enhances positive experiences compared to a physical exercise class (Keisari et al., 2022). In addition, a qualitative study in DMT has shown that synchronized bodily communication fosters closeness, joy, and vitality, and facilitates creativity and playfulness in shared movement (Shuper Engelhard, 2018). In contrast, movement rhythms lacking coordination can lead to feelings of disconnection and loneliness. Another qualitative study indicated that movement synchronization can facilitate emotional regulation (Pietrzak et al., 2016). These findings may explain why participants in the PTE + DMT group were able to undergo social experiences that evoked positive emotions alongside negative ones.

Another theme that exclusively emerged in the PTE + DMT group is “past memories.” This finding can be elucidated by the active encouragement of the dance movement therapist to integrate memories and associations that arise during movement, as well as by previous findings which indicated that spontaneous movement evoked memories (Capello, 2018). For example, in a study that involved joint dance meetings between grandmothers and granddaughters, some grandmothers had shared life difficulties in the meetings prior to dancing (Shuper Engelhard, 2020). After dancing, these grandmothers subsequently shared positive and pleasant memories, even including those stemming from traumatic experiences such as the Holocaust. The findings of the present study support the suggestion that memory is not solely reliant on cognitive abilities in old age, but that there is also a body memory that can be stimulated through the sensorimotor system (Koch et al., 2012).

The subtheme of “playfulness” emerged from the association of movement with past experiences, specifically childhood memories, and was expressed through playful behavior during the sessions. Playfulness has the potential to alter perceptions by framing or reframing situations in a way that provides amusement, humor, and entertainment for oneself and possibly others (Barnett, 2007). Recognized as an important component of healthy aging in older adults, playfulness contributes to their mental and emotional well-being (Korin et al., 2023; Parker et al., 2023). In the context of falls prevention, a previous study demonstrated that incorporating playful exercises, including games targeting postural control, resulted in improved functional balance compared to a control group receiving care as usual (although not statistically significant, *p* = 0.11) (Ehrari et al., 2020). Furthermore, these playful exercises fostered a joyful social atmosphere, motivating participants to adhere to the exercise program and enhancing overall well-being. However, cultural norms and ageism have had a negative impact on older adults’ willingness to engage in playful thinking and behaviors (Korin et al., 2023). Therefore, the present findings highlight the potential of DMT’s invitation for spontaneous movements during physical exercises to elicit memories and serve as a legitimate means to revitalize positive experiences, encourage playfulness, and cultivate a sense of empowerment.

Overall, the present results suggest that the integration of PTE and DMT promotes a more positive self-perception of aging (SPA; the expectations and attitudes toward an individual’s own aging) and resilience among the participants. DMT, as a strength-based approach, enables individuals to explore their strengths and resources, facilitating personal growth and healing (Goodill, 2005). This approach has the potential to foster resilience-protective factors among older adults, including self-acceptance, a positive perspective on life, independence, and social support (Bolton et al., 2016). The findings indicate that participants in the PTE + DMT group demonstrated positive SPA and resilience. They expressed self-comfort and self-compassion in the face of age-related changes through spontaneous movements and comforting touch. Nurturing self-compassion is essential for addressing challenges related to poor body image and its negative consequences, and it should be cultivated on a daily basis (Thøgersen-Ntoumani et al., 2017). Additionally, participants in the PTE + DMT group displayed an internal locus of control and independence in their capacity to control their bodies and their freedom to rest and seek sources of security and stability. Prior research has shown that a positive SPA enhances physical resilience, which pertains to an individual’s ability to resist functional decline or recover physical health in the face of falls among older adults (Zhang et al., 2023). Therefore, the findings of the current study suggest that the PTE + DMT intervention may reduce the risk of falling by enhancing positive SPA and resilience.

### 4.3. Limitations, further studies and practical implications

This study has certain limitations that must be considered when interpreting the results. These limitations arise from the characteristics of the sample and the nature of qualitative methods. The study primarily included women over 80 years old, many of whom had a history of falls and were at high risk of falling. In future research, it is essential to examine whether similar findings can be obtained by including diverse gender groups, a wider range of age groups within the older adult population, and individuals with varying levels of fall risk. Furthermore, the present study utilized a qualitative paradigm due to the relatively unexplored research topic. It should be acknowledged that the data collected through process diary do not represent an absolute truth; rather, they are influenced by the chosen perspectives. Nevertheless, it is noteworthy that three researchers rigorously analyzed the data to minimize potential biases, and the analysis integrated objective observations. Future research should incorporate quantitative analysis in a larger sample to investigate the effects of a fall prevention intervention that integrates PTE and DMT on the emotional experience of older adults. The subsequent research analysis should include an examination of self-image and sense of belonging, as well as other emotional, social, and physical measures. By doing so, a broader and more comprehensive understanding of the field can be developed.

The current study provides valuable insights into the emotional experiences, strengths, and challenges of the older population, particularly in relation to falls prevention practices. Recognizing, understanding, and facilitating the expression of these experiences may contribute to the well-being and overall health of older adults. Based on the present findings, it is recommended to incorporate an integration of structured instructions based on professional exercise knowledge and open instructions. Open instructions can create an environment conducive to the expression of emotional experiences during physical practice, while catering to the individual needs of the participants. It is important to note that the possibility of expressing negative emotional contents is significant in order to contribute to the experience of acceptance and enhances intimacy, trust, and received support (Ruan et al., 2020). This is accomplished through the establishment of a safe space that promotes the expression of challenging emotions, thus ensuring individuals are not left alone to deal with these emotions. Along with this, it is essential to emphasize the positive emotional contents and strengthen them, and expand the thinking about negative contents. The integration of these instructions allows for the concurrent experience of positive and negative emotions. This integrated approach presents a comprehensive strategy for fall prevention, and ensures the integration of emotional experiences into the lives of older adults. Furthermore, the results underscore the significance of group-based physical exercise in fostering a sense of belonging and support among older adults, which can increase mental health and well-being.

## 5. Conclusion

The current study introduced an innovative intervention aimed at preventing falls in older adults through the integration of PTE and DMT. The PTE only intervention is based on a PT fall prevention program consisting of muscle strength and balance exercises. The instructions provided in this intervention are structured, emphasizing the physiological aspect of exercise performance. The PTE + DMT intervention integrates PT and DMT techniques to address the emotional experience that arises during physical exercise. This intervention integrates structured and open instructions, encouraging participants to engage in spontaneous and creative movements. Moreover, participants are invited to share their feelings, sensations, and thoughts verbally during the sessions.

The findings demonstrated that in general, exercises focused on balance elicited a range of emotional experiences among older adults, encompassing both positive and negative aspects. These emotional experiences involved verbal and movement expressions that were connected to various aspects of aging, such as (1) self-image and self-worth, and (2) the individual in relation to others. Notably, when the emotional experiences during the exercises were addressed during the PTE + DMT intervention, additional content related to (3) past memories emerged. The additional elements of DMT also enhanced positive perceptions of the self-image, self-worth, and the individual in relation to others aspects, and various displayed abilities.

## Data availability statement

The raw data supporting the conclusions of this article will be made available by the authors, without undue reservation.

## Ethics statement

The studies involving humans were approved by the ethics committee of the University of Haifa, Haifa, Israel (Approval 2699). The studies were conducted in accordance with the local legislation and institutional requirements. The participants provided their written informed consent to participate in this study.

## Author contributions

MPB: Conceptualization, Data curation, Formal Analysis, Investigation, Methodology, Project administration, Writing–original draft. ME-G: Conceptualization, Funding acquisition, Methodology, Supervision, Writing–review & editing. ESE: Conceptualization, Funding acquisition, Methodology, Supervision, Writing–review & editing.

## Funding

The author(s) declare financial support was received for the research, authorship, and/or publication of this article. This research has been funded by the Emili Sagol Creative Arts Therapies Research Center (CATRC) at the University of Haifa, and Institutional Excellence Scholarships for Outstanding Doctoral Students Academic Year 2022–2023 Graduate Studies Authority.

## Conflict of interest

The authors declare that the research was conducted in the absence of any commercial or financial relationships that could be construed as a potential conflict of interest.

## Publisher’s note

All claims expressed in this article are solely those of the authors and do not necessarily represent those of their affiliated organizations, or those of the publisher, the editors and the reviewers. Any product that may be evaluated in this article, or claim that may be made by its manufacturer, is not guaranteed or endorsed by the publisher.
